# Thresholds for the presence of glacial megafauna in central Europe during the last 60,000 years

**DOI:** 10.1038/s41598-022-22464-x

**Published:** 2022-11-21

**Authors:** Frank Sirocko, Johannes Albert, Sarah Britzius, Frank Dreher, Alfredo Martínez-García, Anthony Dosseto, Joachim Burger, Thomas Terberger, Gerald Haug

**Affiliations:** 1 Institute for Geoscience, Johannes Gutenberg-University, Mainz, Germany; 2 Max Planck Institute for Chemistry, Mainz, Germany; 3 Wollongong Isotope Geochronology Laboratory, School of Earth, Atmospheric and Life Sciences, University of Wollongong, Wollongong, NSW Australia; 4 Institute of Organismic and Molecular Evolution (iomE), Palaeogenetics Group, Mainz, Germany; 5 Göttingen, Seminar for Pre- and Protohistory, University of Göttingen, Göttingen, Germany

**Keywords:** Climate sciences, Environmental sciences

## Abstract

Lake sediment records from Holzmaar and the infilled maar of Auel (Eifel, Germany) are used to reconstruct landscape changes and megafauna abundances. Our data document a forested landscape from 60,000 to 48,000 yr b2k and a stepwise vegetation change towards a glacial desert after 26,000 yr b2k. The Eifel landscape was continuously inhabited from 48,000 to 9000 yr b2k by large mammals, documented by the presence of spores of coprophilous fungi from *Sordaria* and *Sporormiella* fungi that grow on fecal remains of the megafauna. Megafauna reached higher numbers during cold stadial climates but was present also during the warmer interstadials. Highest abundance was at 56,500/48,500/38,500/33,000/27,000/21,000/16,200/14,000 yr b2k, i.e. under different climate regimes. Some of these dates were associated with clear human presence, which indicates that megafauna was not overkilled by humans. In contrast, human presence could quite likely have been stimulated by the abundant food supply. Megafauna presence decreased significantly when tree abundance increased during interstadials. The Megafauna disappeared finally at 11,400 yr b2k with the development of the early Holocene forest cover, which appears to be the most important threshold for megafauna presence.

## Introduction

**Figure 1:**
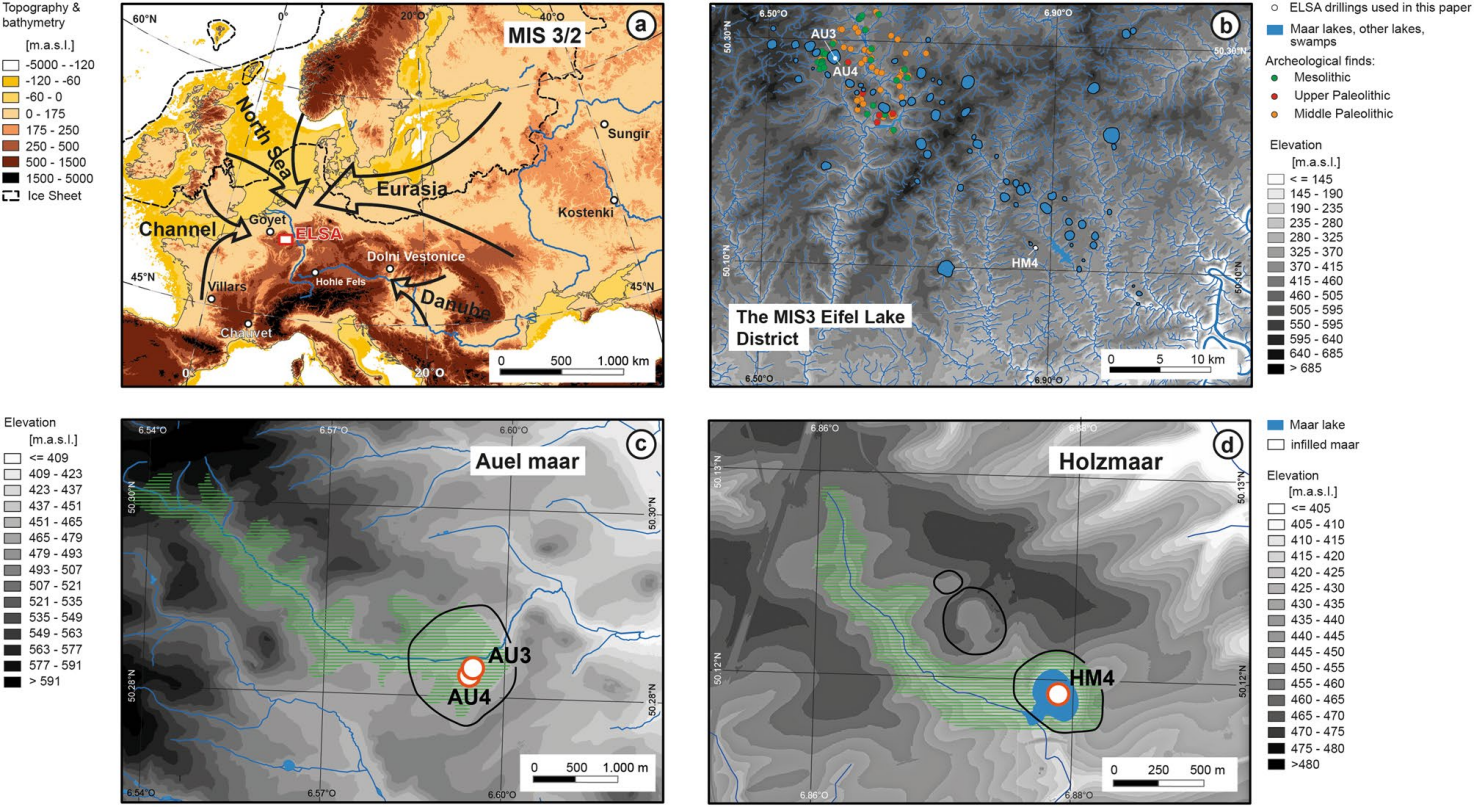
Maps, (**a**) Digital Elevation of Europe with potential corridors for MF migration, (**b**) Maar lakes during the MIS3, (**c**) catchment of the infilled maar lake at Auel, (**d**) catchment of the Holzmaar lake. The maps were made with QGIS 3.16 (https://qgis.org) from ETOPO1 1 Arc-Minute Global Relief Model (NOAA National Geophysical Data Center, 2009), a model of Earth's surface that integrates land topography and ocean bathymetry.

The Eifel is a volcanic field located in the western part of central Europe (Fig. 1a, b). It has a total of more than 250 volcanic structures including 68 maar lakes (60 of which are infilled lakes), which makes it an ideal research field to study the relationships between climate, volcanism, vegetation, landscape and ecology. The sediment cores studied here were drilled by the ELSA-Project, which has systematically cored all accessible Eifel maar sites, lakes and infilled maar structures over the recent years^1–4^, see more information on all ELSA cores on the webseite www.ELSA-Project.de. We use cores from the Holocene maar lake of Holzmaar and the infilled Pleistocene maar lake of Auel, both of which have a similar landscape structure with riverine inflow from catchments of about 5 km length (Fig. 1c, d). These large catchments are important for our study on the megafauna, because it provides a large area, where large animal herds must have grazed.

Sirocko et al.^1^ recently have presented the stratigraphy for those records – the ELSA-20 time series of C_org_(chlorins)^1^, reproduced in Figs. 4, 5, 6, 7. The samples used for this study are from the same cores, have a thickness of 10 cm, were freeze dried, gently homogenized and subsampled for pollen/spores, alkanes, and lithium isotope analysis. This collection of 1000 samples is presented with this paper as the ELSA-20-Stack. Supplementary Table S1 documents the details about the samples; their exact positions in the cores are also documented in Supplementary Figs. S1–S5.

The ELSA-20-Stack also includes the Holocene, which was analyzed in core HM4 from Holzmaar, 30 km distant to Auel. Holzmaar is smaller than Auel, with a diameter of 325 m, but has also a catchment of 6 km length (Fig. 1d). The samples used for this study are from the same cores, and were subsampled for the analysis of pollen/spores, leaf waxes (*n*-alkanes), and lithium isotopes. Supplementary Table S1 documents sample details, with their position in the cores documented in Supplementary Figs. S1–S5.

**Figure 2:**
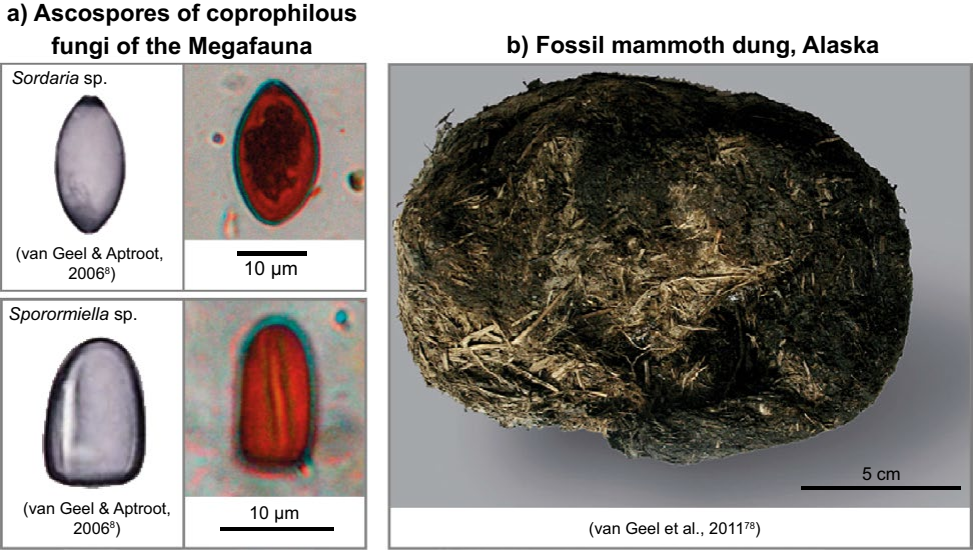
Ascospores of coprophilous fungi, (**a**) photos of spores in textbook^8^ and in the ELSA-Stack samples, (**b**) fossil mammoth dung^78^.

**Figure 3:**
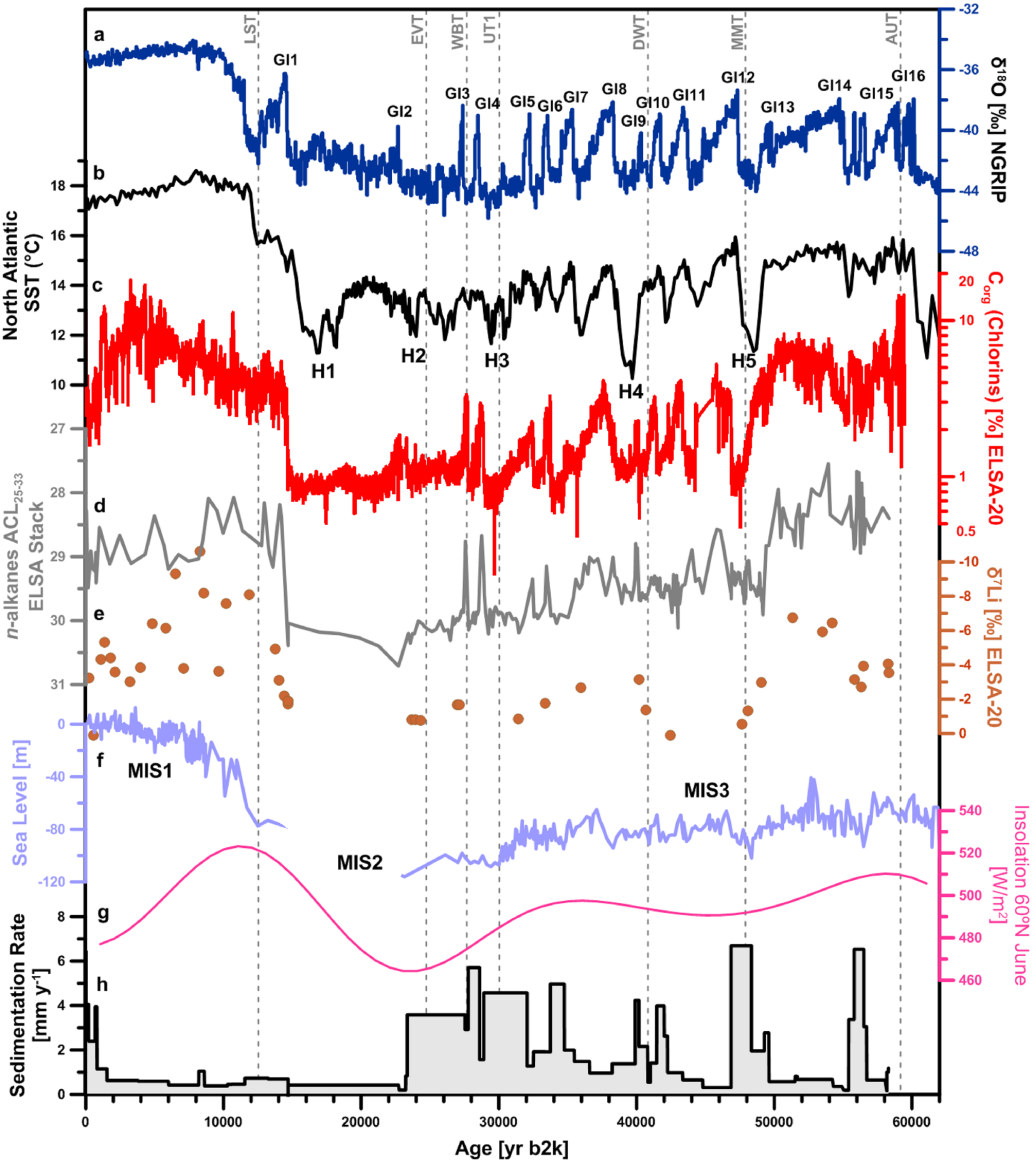
Selected global climate records in comparison to sedimentological and tephra records of the ELSA-20-Stack. Insolation^81^, sea level^16^, North Atlantic Sea surface temperatures^21^, NGRIP δ^18^O^18^, Eifel Tephra^45^.

**Figure 4:**
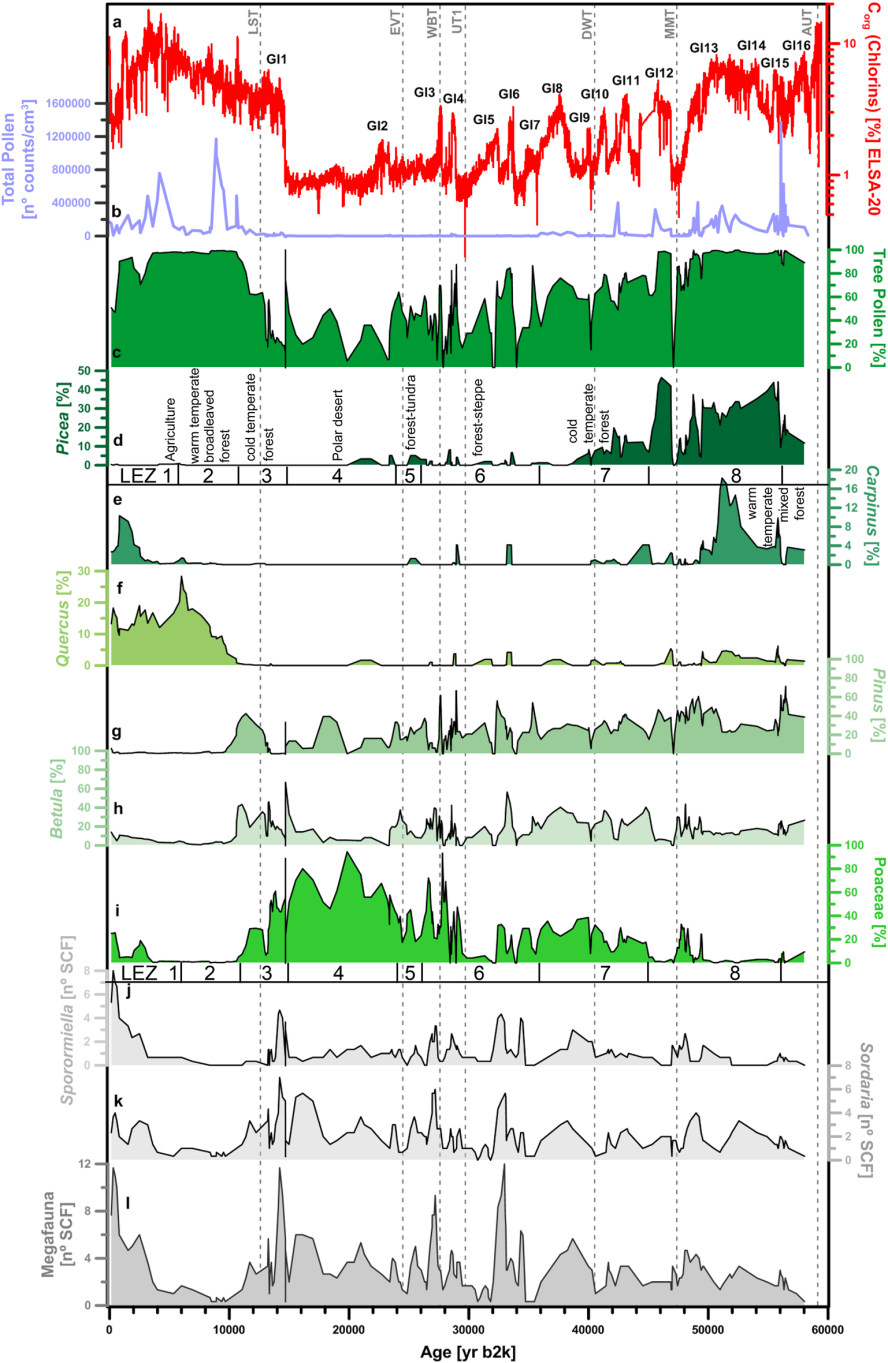
Selected pollen and spores during the last 60,000 years. The plots are based on all samples with countable pollen grains. The curves are smoothed with a three-point running mean. Values for all taxa counted see Supplementary Figs. S8, S9. Landscape Evolution Zones (LEZ)^2^.

**Figure 5:**
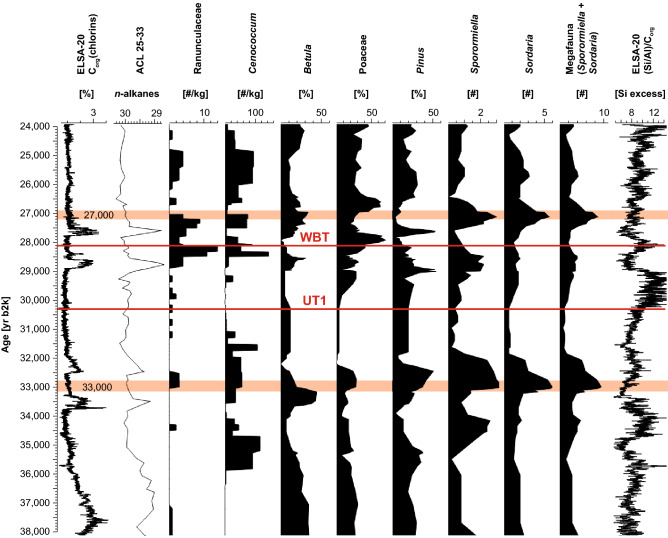
Selected pollen, botanical macroremains, and spores from 38,000 to 24,000 yr b2k. The plots are based on those samples with more than 20 countable pollen grains. The curves are smoothed with a five-point running mean. Values for all taxa counted see Supplementary Figs. S8, S9. Brownish bars indicate the times of high Megafauna presence.

**Figure 6:**
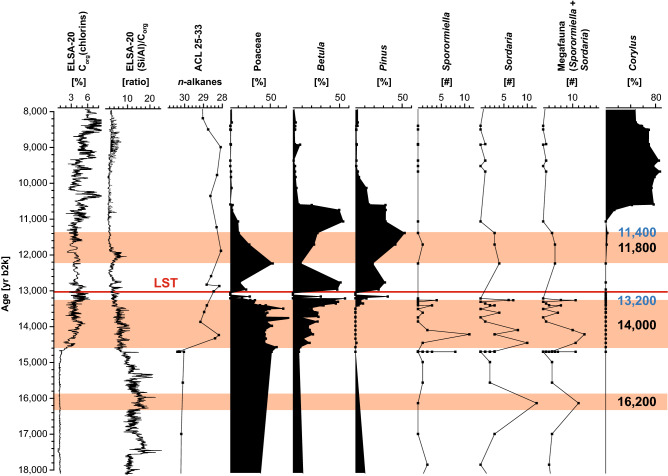
Selected pollen and spores from 18,000 to 8000 yr b2k. The plots are based on those samples with more than 20 countable pollen grains. Values for all taxa counted see Supplementary Figs. S8, S9. Brownish bars indicate the times of high Megafauna presence.

**Figure 7:**
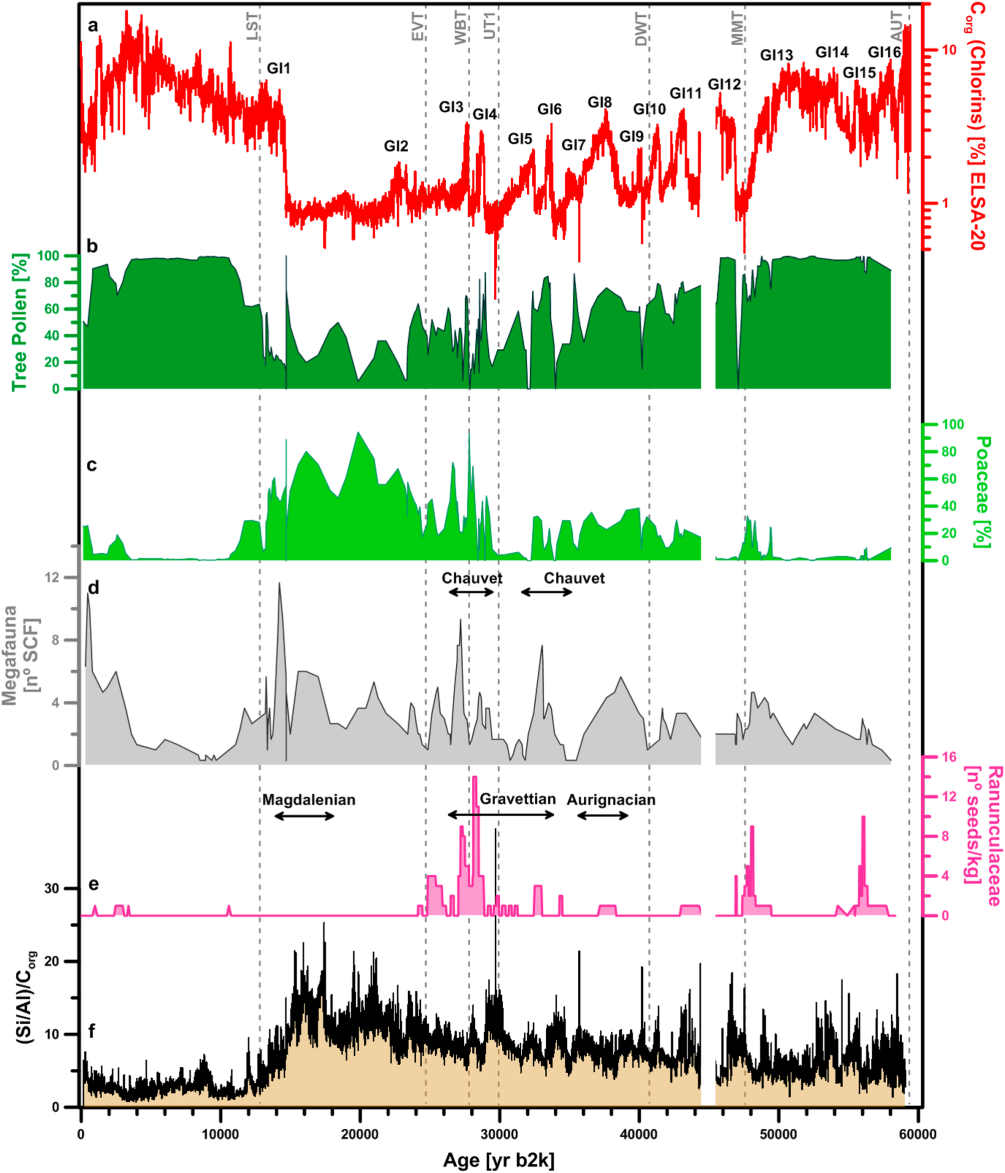
Synthesis of environmental change to explain the presence/absence of Megafauna during the last 60,000 years. The plots are based on all samples with countable pollen grains. All curves are smoothed, C_org_ and Si excess with a 100 year resolution, the pollen and spores to a resolution of 200–500 years.

In this article, we combine the reconstruction of the Marine Isotope Stage (MIS) 3 vegetation in the Eifel (based on pollen, leaf waxes and lithium isotopes), with a study of spores of coprophilous fungi (SCF) from large mammals, which must have grazed in the catchment of the small rivers with inflow into the maar lakes (Fig. 1c, d). We identify the presence of megafauna in the catchment of the lake by analysis of SCF^5–7^, which were counted together with pollen analysis. *Sporormiella* and *Sordaria,* the two most abundant SCF^8^, are regularly found in the sediment from several Eifel maar lakes, but highest spore concentrations were found in the sediments of the infilled maar lakes of Auel and Holzmaar. Using a composite record of SCF (Fig. 2) from both sites, we have studied the presence of megafauna in the Eifel region and compared it to global climate parameters (Fig. 3) and local vegetation (Fig. 4). We present the spore data as counts instead of proportionally as done for pollen data (Figs. 4, 5, 6, 7). This is mainly because spores are present in Last Glacial Maximum (LGM) samples, while pollen is often absent from those samples. However, at least there must have grown grasses, because we see the dropping of grass eating mammals. We attribute this absence to the differential dissolution of pollen in comparison to the thick-walled spores, which apparently survived in the oxygenated deep water of the glacial maar lake. Detailed information on the pollen/spore sample preparation and statistical treatment of the pollen and spore record are given in the Method Section. Spectra of all 32 taxa analyzed are presented in Supplementary Figs. S6–S9. Figures 4, 5, 6, 7 of this main text present only selected pollen. Megafauna includes by definition animals from 45 kg up to 1000 kg^9^. The finding of bones in the caves of Goyet and Spy (Belgium) document the MIS3 mammals in the larger Eifel region in the most detail^10^. Further animal remains are available from the Gravettian open air site of Mainz-Linsenberg in the Rhineland to the west and more distant by Aurignacian and Gravettian layers of cave site in the Swabian Jura to the south^11–13^. Based on such skeletal analyses we know that western central European humans of the MIS3 mostly hunted on (young) mammoth, wooly rhinoceros, bison, and herd animals of lower weight like horse and reindeer. However, these studies lack a fine-chronological resolution. High resolution, well dated European terrestrial records for MIS3/2 times are still scarce and it is thus not possible to evaluate the landscape for all of Europe during MIS3. We thus use a digital elevation model to outline four potential corridors, which could have facilitated long distance migration into the Eifel area (Fig. 1a). One route is along the Danube and Rhine rivers, the second from Eurasia, the other two via the dry North Sea or the Channel regions. All of these regions were subject to intense landscape change during MIS3/2.

The climate along the Danube and the Eurasian corridor has been investigated in detail recently^14,15^. The processes causing landscape change in the two other corridors from the Northwest and West cannot be evaluated precisely, because they were dominated by changes in global sea level^16^. Sea level had dropped in steps from a highstand in the early MIS3 to the LGM low level, which must have had a significant impact on megafauna migrations from the North Sea and Channel corridors into central Europe (Fig. 1a).

Data from 56 natural archives have been compiled recently^17^. They generally outline the stadial/interstadial pattern that is well known from the Greenland Ice Cores^18^. Regional climate signals, however, are difficult to compare between records, especially since ^14^C dating for the early MIS3 is problematic. The annually continuous ELSA-20 C_org_(chlorins) record from Auel and Holzmaar is at the moment the only record in central Europe to cover both MIS3 and MIS2 and is in addition fully correlated with the Greenland GICC05 chronology^1^. Here, we use these continuous and undisturbed maar lake sediments to document the presence/absence of megafauna and its relation to climatic, environmental change, human hunting pressure and volcanism. The effect from volcanic eruptions can be tested because the sediments contain layers of ash from 6 large eruptions, which are well visible in the core photos and which can be used to correlate the Auel cores to other ELSA cores^19,20^.

## Results

### Reconstruction of the Eifel climate during the last 60,000 years

Variations in summer insolation during MIS3 on the northern hemisphere was high in the early and late MIS3, with slightly lowered insolation during the middle MIS3 (Fig. 3). On millennial timescales, North Atlantic sea surface temperatures were characterized by five abrupt episodes of cooling known as Heinrich Events H5–H1, which were linked to the collapse of the Laurentide ice sheet^21^ (Fig. 3). Temperatures derived from Greenland ice reveal that the cold conditions of the last ice age were interrupted by 17 warm episodes, called interstadials (Fig. 3). The ELSA-20 C_org_(chlorins) record shows the North Atlantic stadials/interstadials in perfect match with European temperature variations (Fig. 3). Accordingly, the megafauna (and early humans) in the Eifel lived in a climate regime strongly under North Atlantic control.

The NGRIP-tuned ELSA-20 stratigraphy was used to calculate sedimentation rates in the Auel maar lake, which document soil erosion, thus precipitation and vegetation cover in the catchment of the Eifel maar lakes^16^ (Fig. 3). The maximum sedimentation rate of 7 mm/yr was reached at Auel during some MIS3 stadials, in particular H6 and H5 (Fig. 3). The sediments of these sections are all fine-grained silts and clays, uniform with only few faint laminations about 5 mm thick, thus matching the values of the tuned sedimentation rate. Accordingly, Eifel landscapes received intense precipitation during stadials. The fine-grained uniform lithology of the stadial sections suggests that precipitation was dominated by summer rains. Winter meltwater events (flood layers) were prominent only during times free of vegetation during several of the Heinrich events^23^. The early MIS3 stadials were thus not perennially arid, but MIS3 rain, which we locate mainly in the frost-free summer and fall months, transported eroded clay particles into the lakes.

Stadial spring, however, could have been quite arid, indicated by the deposition of loess at many sites in Europe, but also by the presence of eolian material in the lake sediments. The ELSA cores have been studied for eolian dust proportions, which were high during stadials, in particular during Heinrich events^24–27^. We present in Fig. 5 a new approximation to characterize excess silicon (Si) in the Auel cores on an annual basis, directly comparable to the climate time series of the ELSA-20 C_org_(chlorins) and Si/Al records. To approximate the eolian Si proportion of the bulk Si record, we normalize the two diatom proxies (C_org_ and Si/Al) to a C_org_/(Si/Al) ratio, which separates the diatom silicon from those silicon proportions from clastic mineral grains like quartz. The C_org_/(Si/Al) ratio (excess Si) time series indicates the proportion of quartz-bound Si, which is from eolian dust during glacial times^28^. This dust proxy varies with the stadial to interstadial rhythm and increases continuously from the early MIS3 to the LGM, when it reached highest values after 26,000 yr b2k and documents the maximum dust content in the Auel sediments during the LGM (Fig. 7).

Another important aspect of palaeo-environmental reconstruction is soil development and how it relates to changes in climate and vegetation cover. Past changes in soil development can be derived from the lithium isotope composition of sediments (noted δ^7^Li), which documents clay formation^29,30^. Lower δ^7^Li values indicate extensive clay formation, and by proxy suggest landscape stability (i.e. decreased soil erosion) and soil development. The forested millennia of the early MIS3, which coincides with wet climatic conditions, is associated with active soil formation, as illustrated by negative δ^7^Li values in the Auel sediments (Fig. 3)^29^. This can be explained as a vegetation cover dominated by trees would promote landscape stability^31^ allowing for active soil development. After about 50,000 yr b2k, δ^7^Li values increase, possibly reflecting active erosion and resulting in the sediments deposited being derived mostly from remnant, poorly-developed soils. For the remainder of the Pleistocene, δ^7^Li values remain relatively high (close to values expected in poorly-developed soils) except for a negative excursion coinciding with Greenland Interstadial 9 (GI9). This observation indicates that prevailing dry conditions between 45,000 and 15,000 yr b2k resulted in poorly developed soils. Negative δ^7^Li values are only observed again between 6000 and 2000 yr b2k, coinciding with warmer and wetter conditions, and a vegetation cover dominated by trees.

### Reconstruction of the Eifel vegetation by analysis of pollen, botanical macroremains and leaf waxes

The analysis of pollen, botanical macroremains and leaf wax compounds (long-chain *n*-alkanes) in the ELSA-20 record indicate that the abrupt temperature changes recorded by lake productivity tracers were coupled to major changes in structure of terrestrial ecosystems. The distribution and isotopic composition of long-chain *n*-alkanes can be used to reconstruct past changes in vegetation. Although there is considerable interspecific variation, C4 grasses tend to synthetize *n*-alkane with a maximum around C_31_ and a relatively high proportion of C_33_, while C3 trees and shrubs tend to show a maximum around C_29_ and a relatively higher proportion of C_27_^32,33^. The average chain length (ACL) index can be used to express changes in the carbon number of the most abundant *n*-alkane homologue^34^. In general, in the ELSA-20 record, the ACL of leaf waxes approaches values typical of C3 trees, shrubs and grasses during warm periods of the Holocene and interstadials of MIS3, but show values characteristic of C4 grasses during cold periods (i.e. LGM and most MIS3 stadials) (Fig. 3). Althought, the species of C4 grasses could not be identified, the *n*-alkane pattern is in good agreement with the long-term changes in vegetation types recorded in pollen assemblages, which show a clear dominance of grass pollen from the family Poaceae (which includes C4 grasses) during cold periods.

The time series of pollen reveals a forested landscape dominated by spruce (*Picea*), oak (*Quercus*), hornbeam (*Carpinus*) from 60,000 to 48,000 yr b2k (Fig. 4, Supplementary Fig. S9). This phase terminated abruptly with the North Atlantic cold event Heinrich 5 (H5) at 48,000 yr b2k, when hornbeam disappeared, which indicates a sharp drop of Mean Annual Air Temperature below 5°C^35^. The pollen evidence is matched by an increase in the ACL of *n*-alkanes, which suggests an abrupt drop in C3 vegetation, and an increase in C4 grasses at 48,000 yr b2k. These changes may be linked to seasonal changes in precipitation, as well as changes in summer temperatures during cold periods (Fig. 3). Many samples of the subsequent millennia, i.e., during H5, are pollen sterile, while the sedimentation rate was at a maximum. Accordingly, H5 was a cold and wet stadial (see above), with vegetation almost absent. The Li isotope record (Fig. 3) shows increasing δ^7^Li values between ca. 50,000 and 47,000 yr b2k, indicative of a possible stripping of the well-developed soils of the early MIS3, followed by little soil development consistent with an absence of vegetation. The pollen assemblages of the interstadials after H5 (GI12, 11, 10 and 9) are characteristic of an open woodland, termed Landscape Evolution Zone (LEZ) 8 in Sirocko et al.^2^.

We observe another extreme cold phase at 43,000 yr b2k, when the sediments of five different maar lakes show indications of frozen sediment relocation, well visible in the Auel record^1^. This extreme cooling event will be presented in an upcoming publication. While this cold phase could have been significant for the migration of the early Aurignacian humans into central Europe, it did not affect megafauna presence. Megafauna survived also the time of the Campanian Ignimbrite at 39,800 yr b2k and the subsequent H4 cold event, which was also characterized by high sedimentation rates at Auel, and was thus again cold and wet, but with at least some vegetation and thus megafauna presence. The vegetation of the subsequent GI8 is similar to that of preceding GI12–9, however, tree pollen was low and spruce disappeared, while grass pollen further increased. This time period falls into the interval of the lowest summer insolation of the middle MIS3 (Fig. 3).

Insolation on the northern hemisphere increased again after 35,000 yr b2k (Fig. 3) and we observe an increase of all trees during GI7–3 (Fig. 4, Supplementary Fig. S9). In particular GI4 and GI3 reveal a clear spike in the *n*-alkane ACL record, indicating that these interstadials had again high tree abundance. Lithium isotope compositions show poor soil development during this period (Fig. 3). It is possible that despite tree cover increasing, it did not reach the threshold value allowing for landscape stability and soil development as observed during the early MIS3 and the mid Holocene. This threshold value in tree cover needs to be exceeded in order to achieve landscape stability and thus significant soil development to take place^31^. These interstadials appear to have had warmer summers than the preceding GI14–GI8; an observation, which cannot be explained by orbital controlled insolation. Instead changes in the North Atlantic Meridional Overturning Circulation (AMOC) must be invoked to explain the temperature anomalies of these warm interstadials^1^.

The landscape after GI4 was a tundra, well documented by seeds of terrestrial Ranunculaceae (Fig. 4). The tundra (LEZ 5) terminated at about 25,000 yr b2k (Fig. 4) and the landscape turned into a cold desert with frequent dust, a situation that intensified after 23,500 yr b2k^26^. The subsequent LGM was dominated by grass pollen and some moss macroremains; ostracods were apparently very abundant in the cold and well oxygenated water of the LGM maar lakes. Many samples of the LGM are pollen sterile, either because of a temporary absence of vegetation or a complete dissolution of pollen in the well-oxygenated water of the LGM maar lake. Pollen of subarctic taxa is observed in very low count numbers and show only grass, pine and birch pollen. These observations are consistent with the high ACL values of *n*-alkanes, which indicate that under coldest conditions the vegetation was dominated by C4 grasses.

The abundance of grass decreased after 16,000 yr b2k but continued to be the main vegetation during and even after the first North Atlantic warming starting at 14,700 yr b2k (Fig. 4). Birch, pine and willow increased slowly in the catchment of Auel and Holzmaar, which fits the vegetation development documented in other Holocene pollen records from the Eifel maar lakes^36,37^. It was around 13,300 yr b2k that pine and in particular birch developed for the first time into a more dense forest^38^. Between 25,000 and 15,000 yr b2k, high δ^7^Li values suggest that sediments delivered to the lakes derived from poorly-developed soils, a consequence of sparse vegetation and dry conditions. It is not until dense forest developed that δ^7^Li values decrease, possibly indicative of landscape stability and soil development.

Grass, with some pine, birch, juniper, and willow characterize the subsequent Younger Dryas (YD). The second abrupt deglacial warming at 11,600 yr b2k was followed by several centuries of decrease in all late pollen of subarctic taxa, but an increase in hazel, which became visible in the pollen record from 11,200 yr b2k onwards (Fig. 6). First presence of hazel was followed by increasing numbers of oak and other thermophilous broadleaf trees, which formed an early Holocene forest cover (see Supplementary Fig. S9). This extensive tree cover would have contributed to landscape stabilization, allowing for soil development, as illustrated by low δ^7^Li values from 6000 to 2000 yr b2k (Fig. 3).

### Reconstruction of the presence of megafauna by analysis of spores from coprophilous fungi

The presence of megafauna is documented by the occurrence of spores from *Sporormiella* and *Sordaria*, which occur in the Auel record for the first time during the stadial climate conditions from 59,000 to 56,000 yr b2k, when the spruce forest turned into a tundra for several centuries (Fig. 4). Which of the four migration corridors the animals used to migrate into central Europe is not known, in any case they moved into a landscape with abundant terrestrial Ranunculaceae, thus most likely a tundra-like environment. The spruce trees characterizing all the rest of GI17–14 disappeared during this tundra phase, and it is only during the tundra phase that megafauna appears in the sedimentary record of Auel (Fig. 4). The signal is best visible in the *Sporormiella* record as a small solitary maximum (Fig. 4); the respective megafauna disappeared promptly with the spruce reforestation after about 1000 years, highlighting the prime role grassland (absence of trees) played for megafauna presence.

The next appearance of the megafauna in the sediment record was with the cooling event at 48,000 yr b2k, after which high numbers of SCF (a proxy for megafauna) appear in the Auel record during the wet and cold millennia of H5. Megafauna presence during the subsequent GI12–11 time period is difficult to quantify, because sedimentation was disturbed in the Auel lake, and all other lakes of this time period, shortly after GI12. Megafauna was however clearly present during the subsequent geomagnetic Laschamp excursion at 42,500 yr b2k. Its abundance was not strongly affected by the Campanian Ignimbrite at 39,500 yr b2k, and it was also present during the H4 event at 39,000 yr b2k, which was again cold and humid.

The 3000 year-long GI8 record reveals taxa as before (Fig. 4), but other trees like *Ulmus* increased also in abundance (see Supplementary Fig. S9). Megafauna was present during GI8, but not in high abundance. It was only after 35,000 yr b2k that *Sordaria* and *Sporormiella* increased. The only parallel vegetation change is an increase of *Cenococcum*. The sclerotia of this ectomychorrhizal fungus form on the roots of a variety of plants on both hemispheres and all climates from the arctic to the tropics^39^. The coincidence of *Cenococcum* and high megafauna abundance is best explained by the decrease of vegetation cover—mainly trees—which caused higher erosion rates into the lake. The most pronounced megafauna maximum develops during the stadial after GI7, and synchronous with the well-dated terminal first phase of art painting in the French Chauvet cave^40^.

The next maximum of SCF abundance is with three spikes between 29,000 and 27,000 yr b2k. The landscape was a tundra during these millennia, but it is again a time of active cave art creation at Chauvet. One explanation for this coincidence could come from environmental changes in the potential migration corridors leading into central Europe; probably the most likely mechanism to synchronize megafauna and human migrations. These millennia witnessed the global fall of sea level at the MIS3 / MIS2 transition. The connection between England and the European mainland developed during this time. Simultaneously, the landscape of Scandinavia and Russia must have changed in front of the expanding continental ice sheets, which were recently dated to the stage of the “Brandenburger ice advance”, after 30,000 yr b2k^41^. A well-known archeological site for the millennia after GI4 is the Czech Site of Dolní Věstonice, which is at the southern Russian corridor, where mammoths were hunted extensively from 31,000 to 29,000 yr b2k^42,43^.

The last Ranunculaceae seeds that documented a tundra landscape disappear at 25,000 yr b2k, but this did not terminate megafauna presence. In contrast, both *Sordaria* and *Sporormiella* reached maximum values after the LGM, when only grass was present and dust activity increased with a change in the main wind direction, reconstructed from the Dehner Maar sediments^26–28^ (see also the new C_org_/(Si/Al) dust proxy; Figs. 5, 6). The LGM section of the Auel maar has many samples with no pollen at all. Either grass had disappeared or the water was so well-oxygenated that pollen dissolved. If pollen was indeed dissolved in oxygenated water, grass and megafauna could have been present continuously from 24,000–13,300 yr b2k. Megafauna was thus possibly present during all of MIS2, but the relative proportion of *Sordaria* and *Sporormiella* changed at 16,000 yr b2k, possibly related to an increase of horse presence, which was the most abundant hunting prey at Gönnersdorf (80 km distant to Auel) at that time^44^.

Surprisingly, the first strong warming episode of the North Atlantic at around 14,700 yr b2k had no strong effect on the Eifel landscape and megafauna presence. Organic matter in the lake sediments increased with warming, probably due to the development of a summer stratification and the development of a seasonal suboxic deep water; but the environment around the lake did not change immediately. Grass persisted throughout the first centuries of the deglacial, and only scattered juniper characterized the immediate changes in the landscape at 14,700 yr b2k^37^. Apparently, the late glacial herbivore herds expanded as long as grass was present. The higher temperature could have even encouraged the growth of herds, because it would have increased the length of the summer season.

The megafauna presence decreased with the development of the deglacial birch and pine forest, in particular after 13,400 yr b2k (Fig. 6), i.e., during the development of the first birch forests, which mainly affected the *Sporormiella* record. The megafauna responsible for this spore did not return to its glacial presence during the subsequent YD. *Sordaria*, possibly representing reindeer, reached a final maximum during the YD, when trees decreased, but did not disappear, whereas grasses spread. Both spore types further decreased with the spread of pine and birch during the early Preboreal (11,600–11,000 yr b2k). It was however not until 10,740 yr b2k that *Sordaria* was reduced to a background level (Fig. 6). Both types of SCF were absent during the Mesolithic times of the early Holocene Mixed Oak forest, but returned with the deforestation (spread of grass) during the onset of Neolithic landscape use (Figs. 4, 7), probably representing cattle in the opening woods and later on meadows.

## Discussion of processes controlling the occurrence of megafauna

An analysis of the impact of climate and environment on the mammals and early human cultures in central Europe must take into account that fire and ash from volcanoes can have a strong impact on a landscape. If a tephra is rich in potassium, the ash affects soil formation and the vegetation positively; whereas primitive ash compositions, like in the Eifel, are non-fertile. In addition, human presence can be interrupted by explosive volcanic eruptions. The tephra record of the ELSA-Tephra Stack^45^ (Fig. 3) shows that volcanic activity had no lasting effect on the vegetation and animals that lived in the Eifel over the past 60,000 years.

Generally, it is assumed that periods of warming may have had a strong effect on the occurrence of megafauna. This hypothesis is not supported by the observation that grass persisted throughout the warming event at 14,700 yr b2k and also that the spore record was not affected by the North Atlantic warming. An increase in temperature alone apparently did not strongly change landscape structure. However, warming is clearly documented in the lake sediments by the increase of C_org_(chlorins) content at that time, which must have been caused by the development of thermal lake stratification during summer, causing deep anoxia and thus an increase in organic carbon preservation.

Cold temperatures were no more a problem for the megafauna than warming periods, as they survived even the coldest conditions of stadials, Heinrich Events and the LGM. Precipitation may not have been a serious challenge for the megafauna either, as shown by the maximum spore concentrations during phases of the earlier MIS3, which are associated with higher sedimentation rates. These high sedimentation rates at Auel are caused by high clay content and were thus apparently a function of the riverine contributions and precipitation. Visible flood layers in the Auel sediments were associated with snowmelt events^23^, which however show no relation to megafauna presence. The general increase of sedimentation rate is most likely due to perennial precipitation, which was apparently high in all stadials. Accordingly, temperature and precipitation alone cannot be regarded as prime factors for megafauna habitats.

Neanderthal Humans are recorded in Europe for the entire period from around 300,000 to 40,000 yr b2k^46^ and must have inhabited the spruce forest of GI17–12. The first appearance of Anatomically Modern Humans (AMH) in Europe might have been as early as 57,000 yr b2k in southeastern France^47^, but in central Europe at that time only Neanderthals were to be reckoned with. The first appearance of megafauna overlaps with late Neanderthals. This is reflected by the presence of spores of both *Sporormiella* and *Sordaria* during the few centuries after GI16, when Ranunculaceae seeds and absence of spruce pollen indicate the development of tundra with herds of megafauna, but only for a few centuries.

The geographically closest evidence for late Neanderthals in the larger Eifel area comes from the Goyet Cave in Belgium from around 40,000 yr b2k^48^. The cave is about 120 km from Auel. There is clear evidence that Neanderthals at that time hunted horses, reindeer, young mammoths and woolly rhinoceroses^48–50^, which we believe to be the mammals that produced the feces on which *Sporormiella* and *Sordaria* grew.

It is still strongly debated when the first Aurignacian people exactly arrived in central Europe. Dates from 43,000 to 37,000 yr b2k have been proposed^51–53^, but without doubt before the long and warm interstadial GI8. Aurignacian people were present in the caves of the Swabian Jura and produced some of the first fully sculptural works of art in human history around 40,000 yr b2k^52^. The AMH living in the Goyet cave are genetically most similar to the AMH living in Kostenki (Russia) and have been described as part of the ancestral European Founder Population^54^. Following the indications as outlined in this paper, they should have come along the Russian corridor and followed the megafauna herds into a landscape where grass started to spread after the millennia of the extreme North Atlantic H4 cold event.

It is however only after GI8 that first Aurignacian cave paintings were dated by both ^14^C and U/Th to 37,000–33,500 yr b2k in the French Chauvet cave^40^. Spore concentrations in the Auel record show low megafauna presence during this time, but the most pronounced spike in the megafauna record is exactly at the end of the Chauvet painting phase. It would be speculative to construct a common mechanism behind this, but a high resolution comparison of the Auel megafauna presence and the terminal painting phase at Chauvet might shed a light on this.

The next episode of cave art at Chauvet is from 31,000 to 28,000 yr b2k, which falls into the Gravettian, starting in central Europe around GI6 at about 34,000 yr b2k and lasted until GI3, at about 27,000 yr b2k^55^. The Auel record shows that these millennia had witnessed spikes of trees, however only during the warm centuries of GI5, 4, 3. These late MIS3 interstadials might have been warmer than the preceeding interstadials GI8–6. All interstadials were related to the intensity of the AMOC^1^, but in addition, GI5–3 fall into a period of increased summer insolation (Fig. 3). It is possible that the North Atlantic AMOC intensification and the general increase of summer insolation were superimposed during the forested interstadials. In between the interstadials, Ranunculaceae were abundant and indicated local or temporary tundra landscape during the stadials, as soon as the AMOC collapsed.

GI4 at 28,000 yr b2k was the warmest of the Gravettian interstadials; spores show high megafauna presence during the interstadial and after the interstadial. It is in line with the observation that human population size in central Europe increased between 43,000 and 29,000 yr b2k and started decreasing only after 28,000 yr b2k^56,57^.

Climate changed drastically at 26,000 yr b2k, well visible in the paleobotanical record at Auel, but also in the subsequent almost complete absence of humans in central Europe. This is a time when humans either migrated south to glacial refugia or, more likely, became extinct in many parts of central Europe. There are archeological arguments against a southward migration^57^, but also genetic ones: demographic modelling of ancient genomes shows that human populations underwent a significant reduction in size around 27,000 yr b2k accompanied by a split into southeastern and southwestern subpopulations^58^. The Auel record shows that some megafauna however remained in the seasonal grassland of central Europe which can be taken as an indication that they were clearly better adapted to the cold than humans (Fig. 7). Little is known about the presence of humans during the LGM in the Eifel region itself. At the site Wiesbaden-Igstadt, in the central Rhineland, and in the Swiss Jura there is sporadic evidence for human presence, which can probably be assigned to GI2^59,60^.

The pollen spectrum and the increased presence of SCF indicate that the Eifel landscape was covered by grass from 16,000 to about 13,300 yr b2k, with trees in increasing numbers after the first Late Glacial warming at 14,700 yr b2k. Both *Sordaria* and *Sporormiella* were abundant until 13,300 yr b2k (Fig. 6) and indicate a constant late glacial megafauna assemblage. *Sporormiella*, however, decreased sharply at 13,300 yr b2k, synchronous with the increase of birch and pine pollen, which formed the first deglacial central European forests. We suspect that mammals like the giant deer *Megaloceros* could not live in a forest and moved into open landscapes like Ireland, where *Megaloceros* reached its maximum population density during the Allerød^61^ when the Eifel, and other parts of central Europe, were apparently covered by a dense birch and pine forest. Following Zimov’s^62^ suggestions, Megafauna may not be affected by vegetational change, but may itself suppress the development of woodland by grasing. Thus, migration of herbivores may result in the decrease of herbs and grasses in favor of tree species. From our data, we can not conclude the exact interplay between Megafauna and vegetation, however, we see a returning pattern of forest closure and disappearance of Megafauna. Strong presence of humans in the Rhineland hundreds of years before the 14,700 yr b2k warming is attested by the Magdalenian sites such as Gönnersdorf and Andernach. The Magdalenians hunted mainly horse and reindeer, while a few mammoth and rhinoceros remains seem to be of sub-fossil origin^63^. During the subsequent period of the Allerød elk, red deer and aurochs was the main prey^64,65^. Sporadic presence of reindeer hunters can be mentioned for the Younger Dryas^66^. The impact of humans on the megafauna in this region during these dramatic climatic and environmental changes cannot be reconstructed at the moment. Ongoing DNA analysis in the Auel sediments might provide some clue into the interrelations between late glacial humans and the megafauna.

## Conclusions

Megafauna abundance shows no relation to periods of active volcanism in the Eifel, suggesting that volcanic activity and associated fire events did not play a role in megafauna extinction. The presence of megafauna does not seem to have been affected by humans either. In fact, megafauna was most abundant in the period from 33,000/27,000 yr b2k, probably fostering human presence of (late) Aurignacian and Gravettian hunters in central Europe. For example, the close connection between the presence of megafauna and humans is documented in the Rhineland for the horse hunter site of Gönnersdorf at ca. 15,500 yr b2k, where it is likely that large herds attracted human hunters. In addition, megafauna inhabited open woodland, steppe, tundra, and even the polar desert of the LGM when only seasonal grass and moss were accessible. Thus, megafauna apparently tolerated all climates, including abrupt warming and cooling events as long as grass grew in abundance.

The main causal mechanism for the decrease and eventual disappearance of megafauna was the development of woodlands. Most likely, as forests grew, the large herbivores lost their main food — grass — and were no longer able to migrate long distances between seasonal grassland regions. The extent to which forests were also an obstacle to a rapid escape from hunters and predators cannot be inferred from the available time series of SCF. Wolves and humans may have had a much greater chance of killing young calves in a dense forest than in an open landscape. In summary, the evaluation of the ELSA-20 stack analysis strongly suggests that forestation was the main factor affecting the presence or absence of megafauna in central Europe during the late Quaternary.

## Materials and methods

### The Eifel maar lakes

The Eifel is located west of the Rhine in Germany; it experienced 200–300 m of uplift during the Cenozoic, leading to the formation of more than 60 Pleistocene maar eruptions. Eight of these maar lakes are today still filled with water, of which six have more than 20-m-deep waters with anoxic conditions at the bottom. All other maar structures are infilled Pleistocene lakes. The largest of these maar lakes were dated by the ELSA Project^2–4^ and erupted during the last 130,000 years^19,20^ (Supplementary Table S1). Mapping of the ejecta from large and small structures indicated that the smaller ones erupted simultaneously with nearby larger structures. According to the ELSA datings of the large Pleistocene maar structures, we expect up to 60 maar structures to have been lakes or swamps during MIS3. Accordingly, we present in Fig. 1b map of the “MIS3 Eifel Lake District” with up to 60 lakes/swamps and numerous creeks (partly dammed by lava flows) that drained towards the Mosel river.

### Palynology

We have analyzed pollen for 250 samples along the entire stack (Figs. 4, 5, 6, 7 of main text, Supplementary Figs. S6–S9). At least two of these pollen samples for each interstadial and stadial have been further analyzed for spores. Each pollen/spore sample spans a depth range of 1 cm and represents a volume of about 1 cm^3^. The sediment was treated with potassium hydroxide solution (KOH), hydrochloric acid (HCl) and hydrofluoric acid (HF). For acetolysis, acetic acid (C_2_H_4_O_2_) and a mixture (9:1) of acetic anhydride (C_4_H_6_O_3_) and sulfuric acid (H_2_SO_4_) was used. Centrifugation was done at 3000–3500 rpm for 5 min. The samples were sieved at 200 µm and later filtered at 10 µm. *Lycopodium*-spore tablets were added for calibration of absolute pollen volumetric concentration. The samples were mounted with liquid, anhydrous glycerol (C_3_H_8_O_3_). Pollen counting was done under an optical microscope at a maximum of 600-fold magnification. Total pollen content (#/ccm) has been calculated using the known number of *Lycopodium* spores in added tablets^67^.

In most samples we were able to count up to 300 pollen grains, however, some stadial samples just show 20 countable pollen grains. 20 counts are statistically problematic, but these samples include often only three taxa (*Pinus, Betula,* and Poaceae). Samples with less than 20 pollen grains have been regarded as being pollen sterile, either because pollen was not produced or dissolved in the oxygen rich stadial and glacial deep water. We document the number of counted pollen grains and also the absence of pollen in Supplementary Figs. S6–S9 and Supplementary Tables S2, S3, S4. In particular the absence of pollen outline a clear pattern; with minimum vegetation during the H5 and H4 events.

In this study, we focused on Holzmaar and Auel sediments, but typical vegetational compositions like during the early MIS3, that is dominated by spruce, are also visible in pollen spectra from other Eifel maar lakes (see Supplementary Fig. S10, Supplementary Tables S5, S6, S7).

### Paleobotanic macroremains

The botanical macroremains for core AU2 had been already published^2^. Time series are not reproduced here, but we include in Figs. 5, 7 the data of Ranunculaceae and *Cenococcum*, which are important to outline phases of tundra vegetation, soil erosion, and the LGM.

Ranunculaceae (crowfoot family) have a worldwide distribution, predominantly in areas with temperate to boreal climates. In the Auel drill core, seeds from Ranunculaceae were abundant during the forest-tundra phase, 29,000–24,000 yr b2k (LEZ 5). Many of the non-aquatic *Ranunculus* species are tolerant to moist soils typical for tundra environments. We therefore use terrestrial Ranunculaceae as the most reliable indicator for tundra vegetation.

### Spores of coprophilous fungi — Indicators of the presence of herbivores

The spore records of *Sordaria* and *Sporormiella* are presented in this study (Figs. 4, 5, 6, 7). Representatives of both genera are coprophilous, which means that they need feces as a substrate. Both taxa are established indicators for the presence of megafauna^5,68–70^.

*Sordaria ascospores are* ellipsoidal with one pore at the top of the spore (Fig. 2)^8^. The spores have been detected on dung of various herbivore species, among them mammoth, moose, and cattle^71–73^. The spores serve as an indicators for Pleistocene megafauna^5,69^ as well as domesticated animals^7,74^.

*Sporormiella* ascospores show three or more septa; the spores fall apart in separate cells, each one with a germ slit (Fig. 2)^8^. *Sporormiella* species are obligate coprophilous and were recorded on feces of, among others, mammoths, cows, rhinoceros, horses, moose, reindeers, and hare^68,72,75–78^. Spore records of *Sporormiella* were used to estimate herbivore biomass flucutations during the Pleistocene^5,68^ and the Holocene^6^.

### Alkanes

Long-chain odd-carbon number *n*-alkanes are important components of the protective waxes that coat the leaf surfaces of almost all land plants. Their insolubility in water, negligible volatility, chemical inertness, and resistance to biodegradation make them excellent biomarker compounds. The distribution and isotopic composition of long-chain *n*-alkanes can be used to reconstruct past changes in vegetation. Although there is considerable interspecific variation, C4 grasses tend to synthetize *n*-alkane with a maximum around C_31_ and a relatively high proportion of C_33_, while C3 trees and shrubs tend to show a maximum around C_29_ and a relatively higher proportion of C_27_^32^. The average chain length (ACL) index can be used to express changes in the carbon number of the most abundant *n*-alkane homologue^34^. C4 grasses have average ACL values of 30.66 ± 0.83, while C3 trees and shrubs show average ACL values of 29.00 ± 0.83^33^. In most environments, estimates of change in vegetation type using ACL agree well with those indicated by its carbon isotopic signature, and correlate with those obtained by independent proxies such as pollen abundances^79^.

Samples were freeze-dried and homogenized and the *n*-alkanes were extracted and separated using an Accelerated Solvent Extractor 350 (ASE). ASE cells (22 mL) were prepared with muffled glass fiber filters, 16 g deactivated silica gel and about 0.5 g of the sediment. The cells were flushed with *n*-hexane for the *n*-alkane fraction. After the extraction, an internal standard (hexatriacontane) was added to the samples to quantify the *n*-alkanes. Subsequently, they were dried in a centrifugal evaporator (‘Rocket’ by Genevac) and re-dissolved in isooctane before analysis. Analysis of the *n*-alkanes was carried out with an Agilent 7890B gas chromatography system with flame ionization detection (GC-FID) using a VF-200 column.

### Lithium isotopes — indicators of soil formation

Lithium (Li) isotopes are used as a proxy for soil formation^30^ and were analyzed for cores AU2 and SM5 (Fig. 3); the data were transferred to the respective stack numbers. The resolution of the Li isotope record is, however, not comprehensive enough to construct a complete stack such as that achieved for pollen and spores.

Sample preparation for Li isotope measurements was undertaken in a Class 10 cleanroom at the Wollongong Isotope Geochronology Laboratory, University of Wollongong, Australia. About 10 mg of ground sediment sample (typically < 63 μm fraction) was dissolved in 48% HF and 65% HNO_3_ at 100 °C for > 12 h. After drying down, samples were re-dissolved in aqua regia at 130 °C for > 12 h to break down any fluorides. Samples were then re-dissolved in 1.5 mL 1 M HCl to perform ion exchange chromatography. Cation exchange columns were calibrated with natural seawater samples. After the chromatography procedure, the Li elution was dried down and taken up in 0.3 M HNO_3_ for isotopic analysis on a MC ICP-MS at the University of Wollongong. Using wet plasma conditions, a 30 ppb single element Li tuning solution yielded a typical intensity of 1 V on ^7^Li, while background was of the order 5–50 mV on ^7^Li. The cones setup consisted of a Ni Jet sampler and X-skimmer and a PFA-100 microflow nebulizer (ESI, Omaha, NE, USA) with a flow rate of 90–150 μL/min with a high sensitivity insert (Thermo Scientific). A standard bracketing technique was applied using IRMM16 as primary standard for ^7^Li/^6^Li ratios. Synthetic standards Li7-N and Li6-N^80^ were used to assess accuracy of isotopic ratio determination. Instrument blanks were measured between each standard and sample by introducing 0.3 M HNO_3_. Blank intensities were then subtracted from each isotope. Corrected ^7^Li/^6^Li ratios were converted to δ^7^Li values using L-SVEC as reference^80^. Results for Li7-N and Li6-N are: δ^7^Li = 30.2 ± 0.3 ‰ (n = 22, 2SE) and -8.0 ± 0.2 ‰ (n = 16, 2SE), respectively. To verify the sediment sample dissolution and ion exchange chromatography protocols for Li isotope measurements, a granitic geochemical reference material JG-2 was processed with every sample batch of 10. The average δ^7^Li value of JG-2 is 0.7 ± 1.0 ‰ (n = 5, 2SE). Measured Li isotope ratios for Li6-N, Li7-N, and JG-2 measured in this study are well within reported values.

Total procedure blanks (n = 3), measured on a Q ICP-MS, yielded 0.8 ng, 0.8 ng, and 0.2 ng of Li. The external reproducibility for Li isotope measurements of natural samples, calculated via the average of the 2-standard error (2SE) of two replicate samples, is estimated at 2.2‰.

### Tephra

All ELSA cores covering the last 60,000 years (Supplementary Table S1) show a total of 7 visible tephra layers, each over 1-cm thick (Fig. 3). The thickness varies at different sites according to the distance to eruption centers and prevailing wind directions. Most of these tephra in the lake sediments were already correlated to known eruptions, namely the Laacher See (13,056 yr b2k), Eltville (24,720 yr b2k), Wartgesberg (28,100 yr b2k), Tephra with unknown eruption center (UT1, 30,300 yr b2k), Dreiser-Weiher (40,370 yr b2k), Meerfelder Maar (47,340 yr b2k), and Auel Maar (59,130 yr b2k)^20^.

All the methods were in accordance with the relevant guidelines and regulations. The permission to collect pollen was given in the drilling permissions.

## Supplementary Information

Supplementary Information 1.

Supplementary Information 2.

Supplementary Information 3.

Supplementary Information 4.

Supplementary Information 5.

Supplementary Information 6.

Supplementary Information 7.

Supplementary Information 8.

## Data Availability

All data is available in the main text or the supplementary materials.
