# Supplementary material for: Thresholds for the presence of glacial megafauna in central Europe during the last 60,000 years
Source: Sci Rep. 2022 Nov 21;12:20055. doi: 10.1038/s41598-022-22464-x (PMC9681729; doi:10.1038/s41598-022-22464-x)
Supplement: Supplementary file 1 — Supplementary Information 1. [file 41598_2022_22464_MOESM1_ESM.pdf]

Supplementary Materials for

Thresholds for the presence of Glacial Megafauna in central Europe during the last 60,000 years

Authors: Frank Sirocko[1*], Johannes Albert[1], Sarah Britzius[1,2], Frank Dreher[1], Alfredo Martínez-García[2], Anthony Dosseto[3], Joachim Burger[4], Thomas Terberger[5], & Gerald Haug[2]

[1]Institute for Geoscience, Johannes Gutenberg-University, Mainz, Germany

[2]Max Planck Institute for Chemistry, Mainz, Germany

[3]Wollongong Isotope Geochronology Laboratory, School of Earth, Atmospheric and Life Sciences. University of Wollongong. Wollongong, NSW, Australia

[4]Institute of Organismic and Molecular Evolution (iomE), Palaeogenetics Group, Mainz, Germany

[5]Göttingen, Seminar for Pre- and Protohistory, University of Göttingen, Germany

Correspondence to: Sirocko@uni-mainz.de

This file includes:

Supplementary Figures S1 to S10

Labels for Supplementary Tables S1 to S7

Other Supplementary Materials for this manuscript include the following:

Data S1 to S7 (separate files)

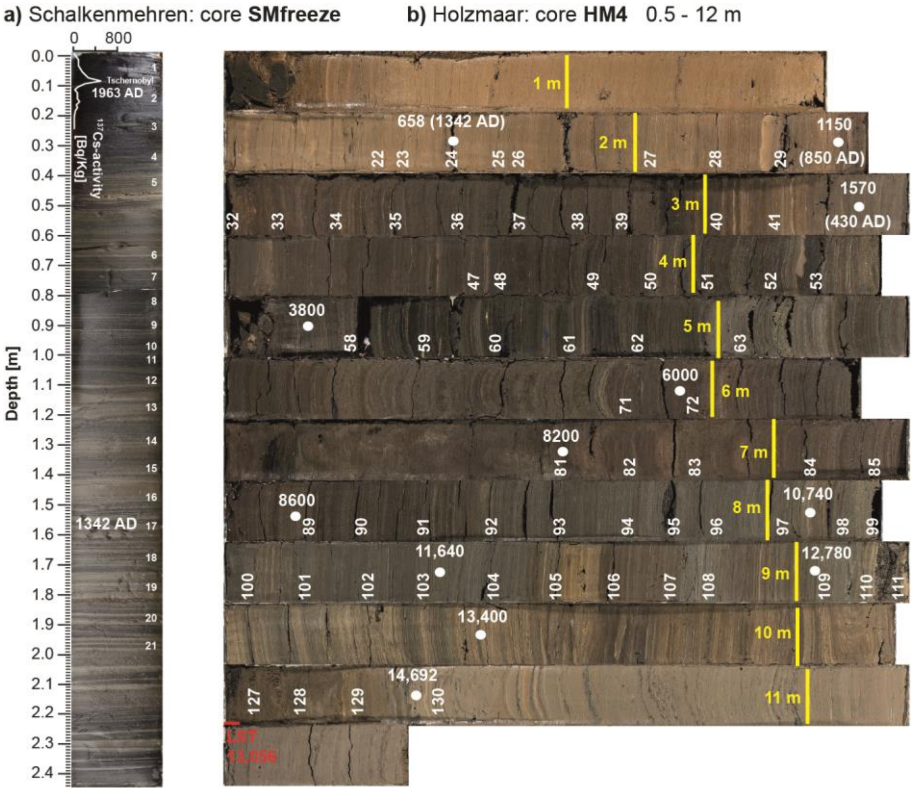

Supplementary Figure S1. Photos of core SMf from Schalkenmehren maar lake and core HM4 from Holzmaar lake. Stratigraphical marker of the ELSA-20 record are documented together with the ELSA-20-Stack sample numbers.

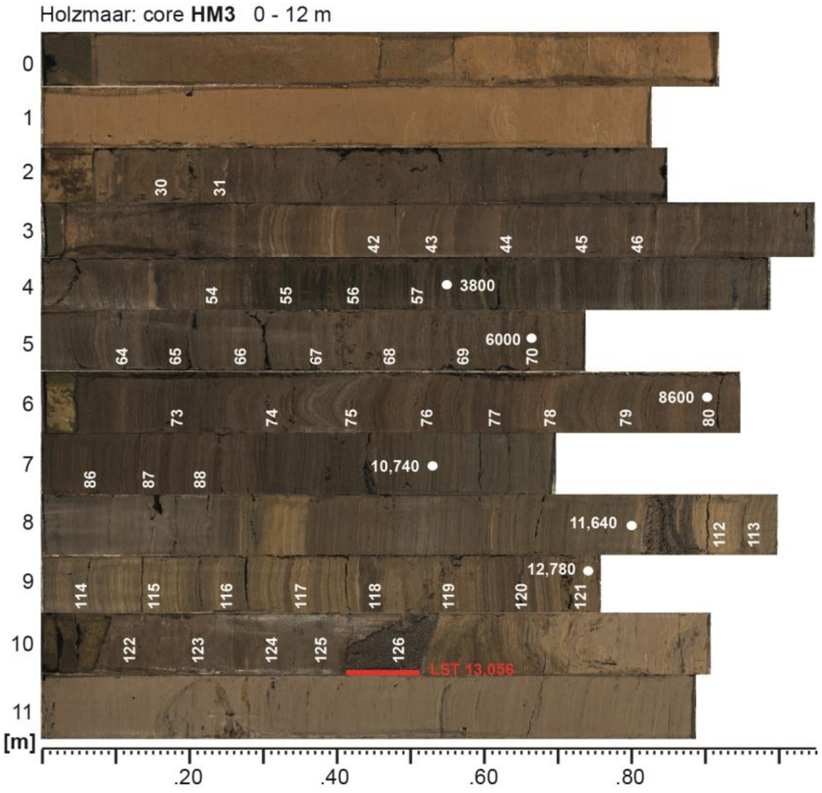

Supplementary Figure S2. Photos of core HM3 from Holzmaar lake. Stratigraphical marker of the ELSA-20 record are documented together with the ELSA-20-Stack sample numbers.

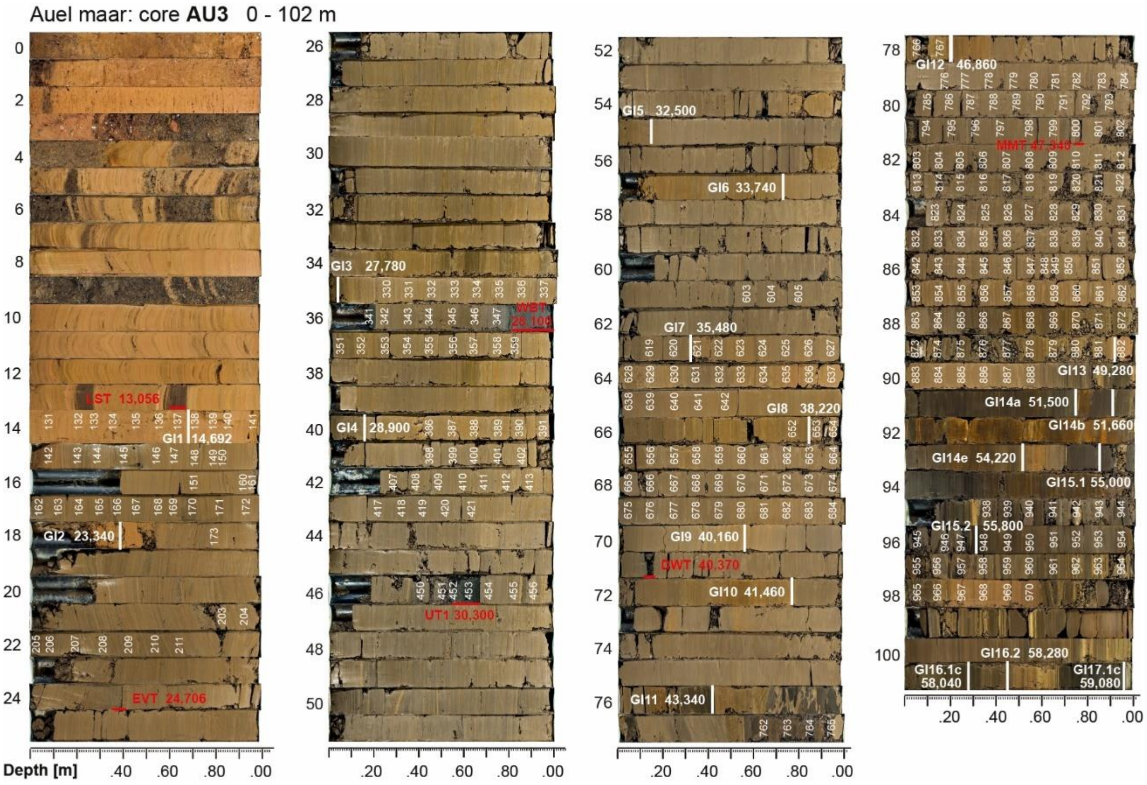

Supplementary Figure S3. Photos of core AU3 from the infilled maar of Auel. Stratigraphical marker of the ELSA-20 record are documented together with the ELSA-20-Stack sample numbers.

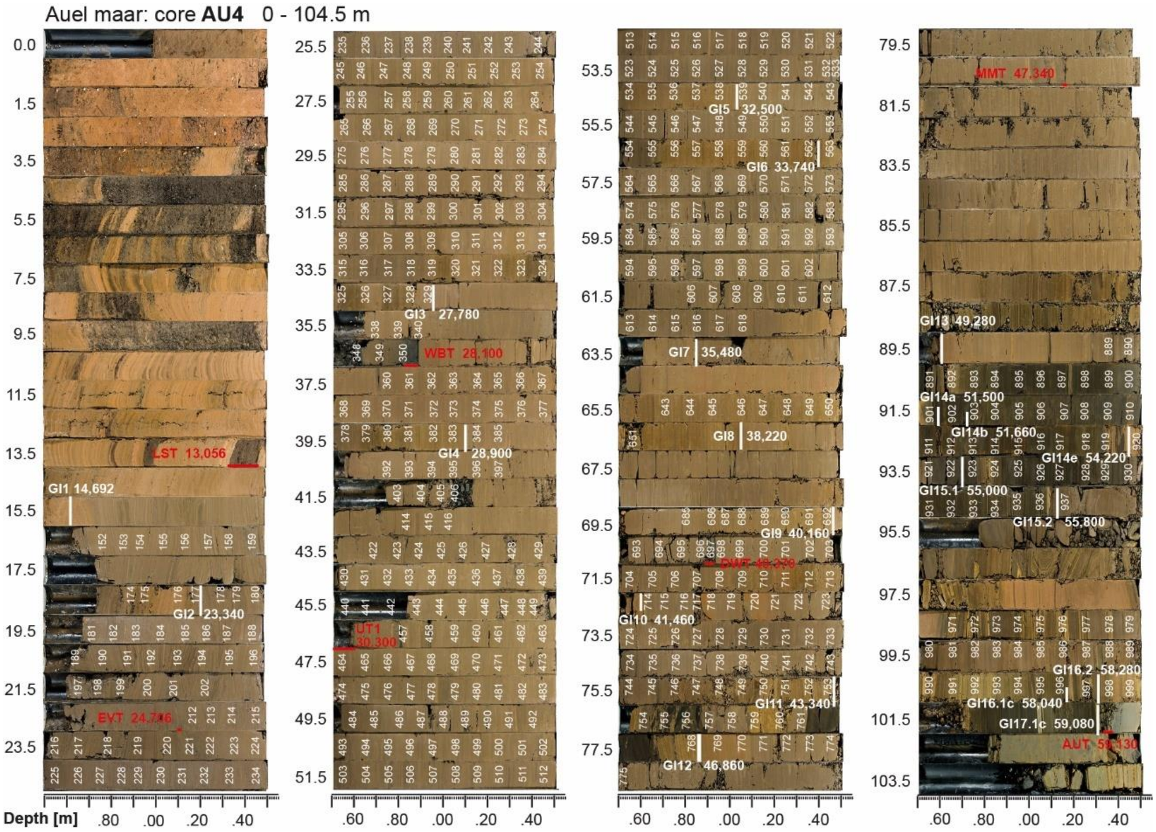

Supplementary Figure S4. Photos of core AU4 from the infilled maar of Auel. Stratigraphical marker of the ELSA-20 record are documented together with the ELSA-20-Stack sample numbers.

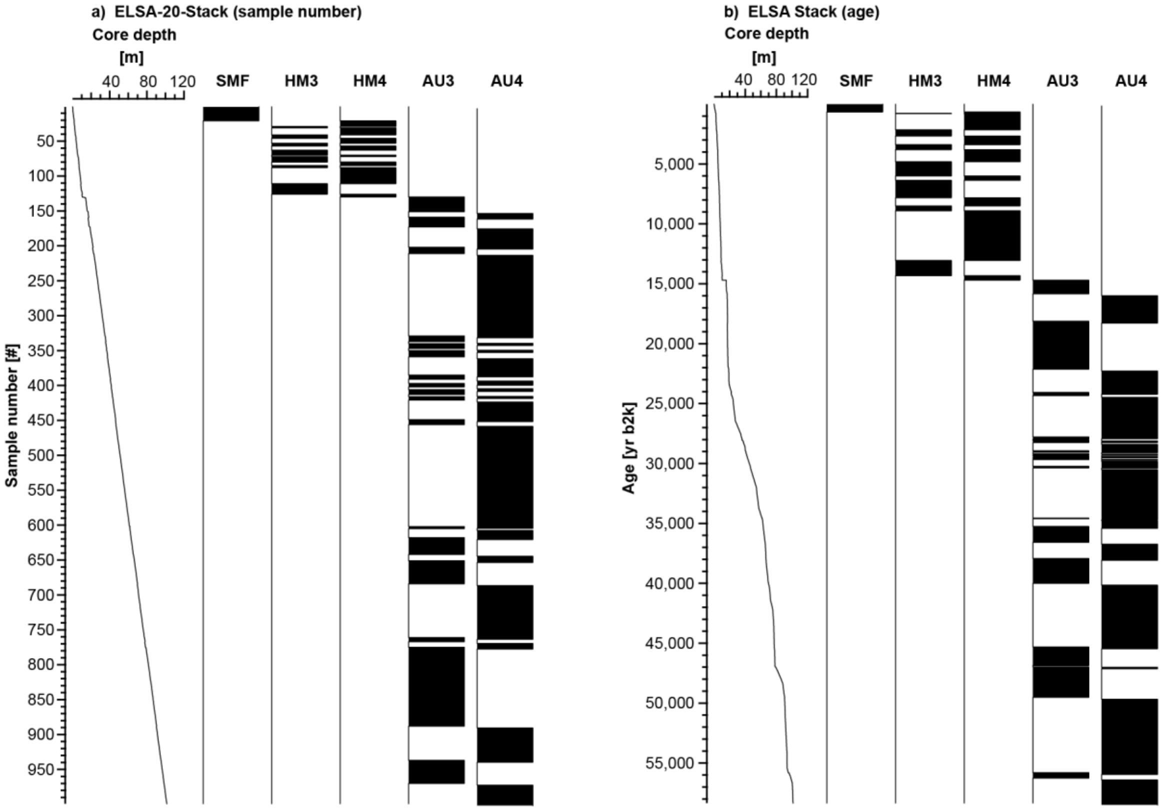

Supplementary Figure S5. Depth of ELSA-20-Stack samples in the sediment cores SMf, HM3, HM4, AU3, and AU4.

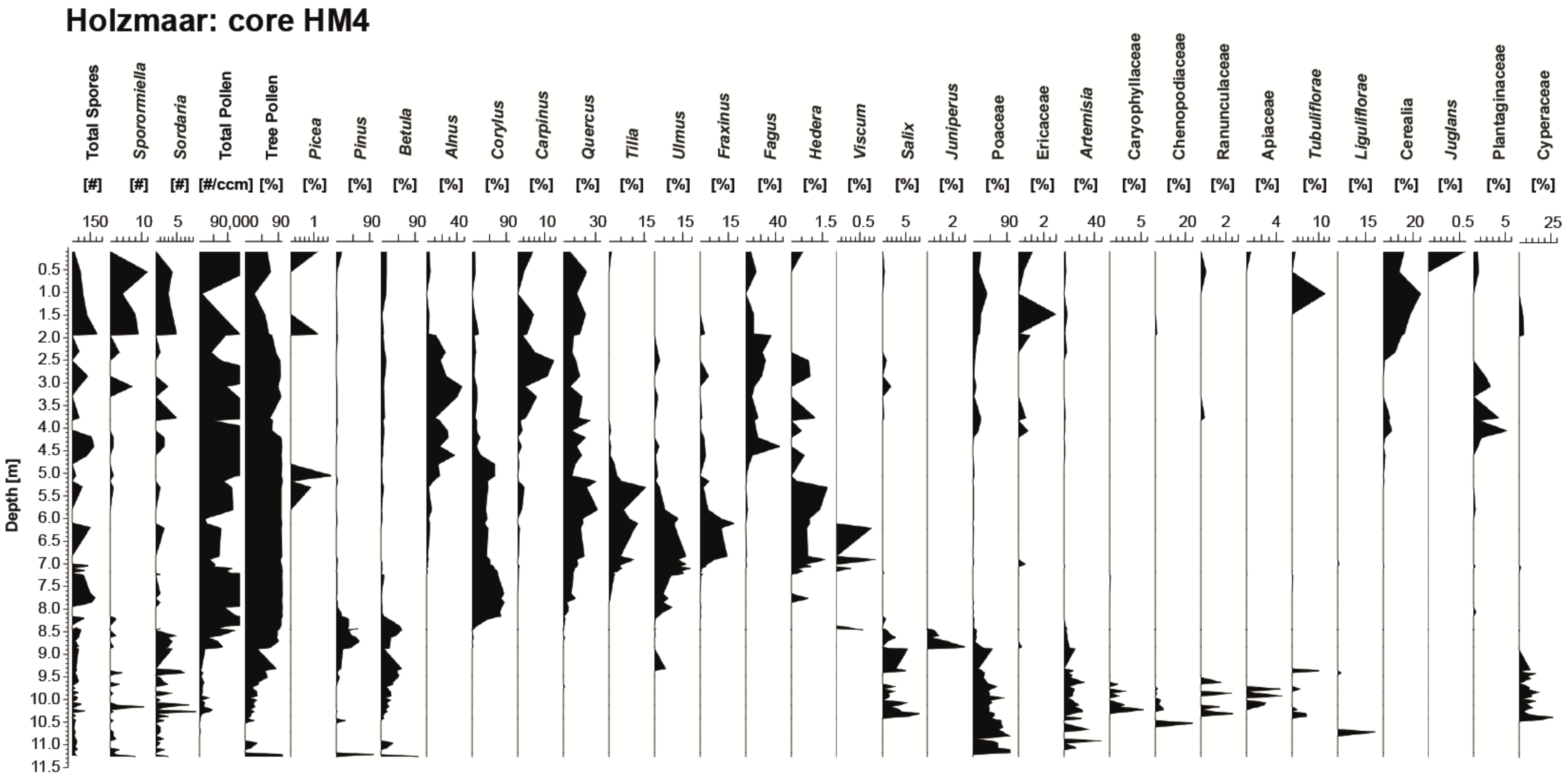

Supplementary Figure S6. Pollen concentrations and spore counts for the HM4 core from Holzmaar versus depth.

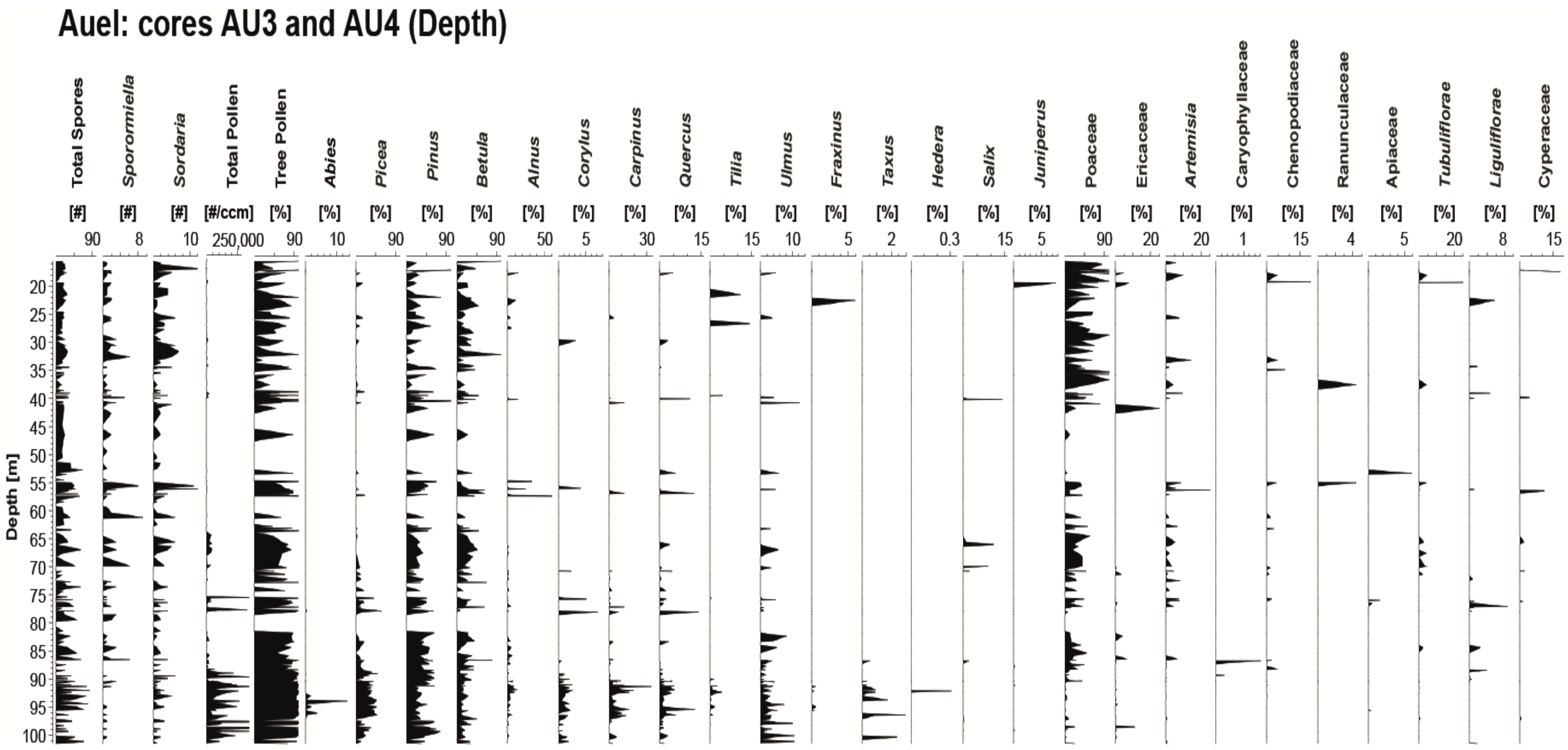

Supplementary Figure S7. Pollen concentrations and spore counts for the AU4 and AU3 core from Auel versus depth

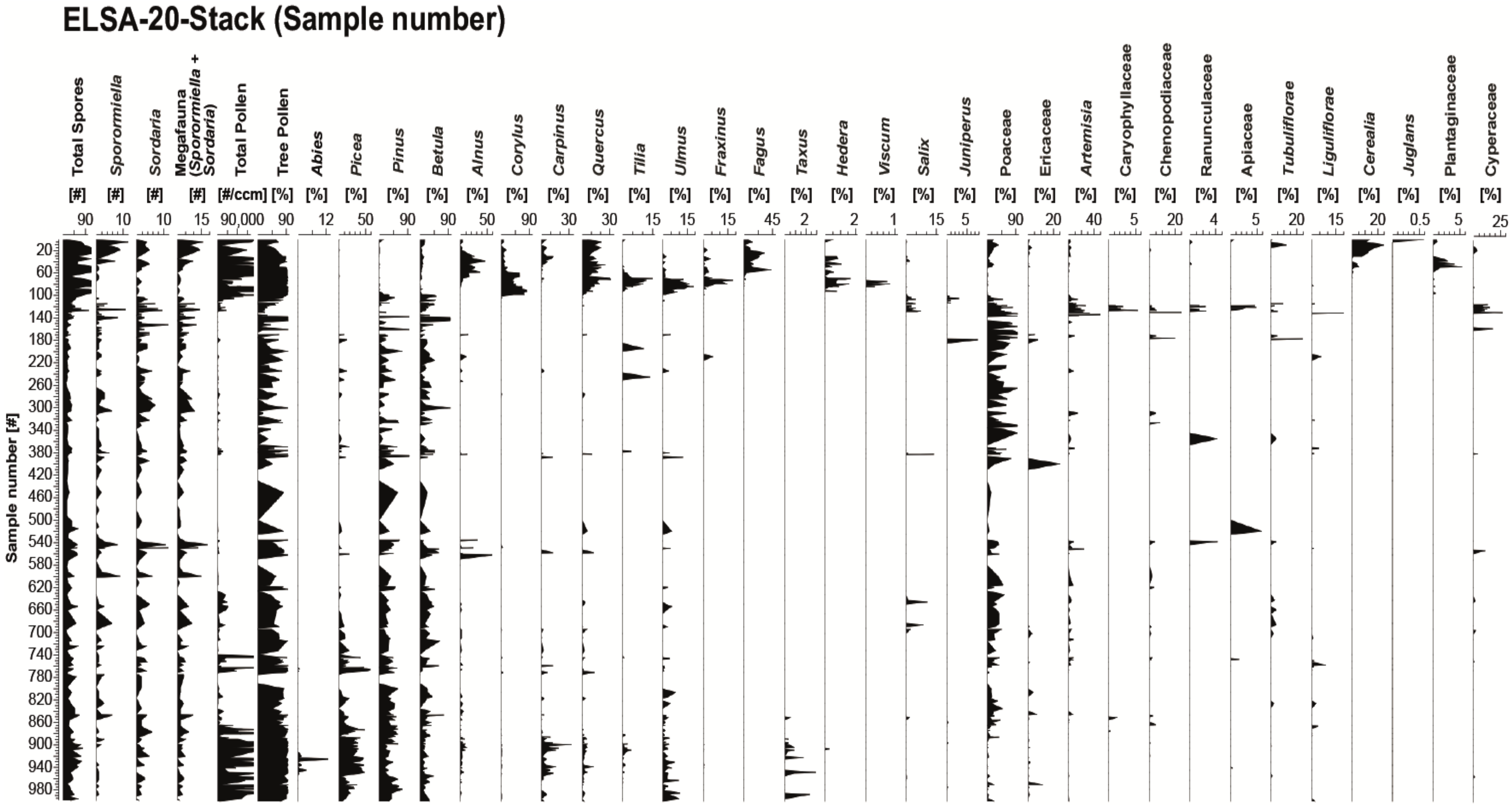

Supplementary Figure S8. Pollen concentrations and spore counts for the ELSA-20-Stack versus Stack number. Shown are only samples with more than 20 pollen counted.

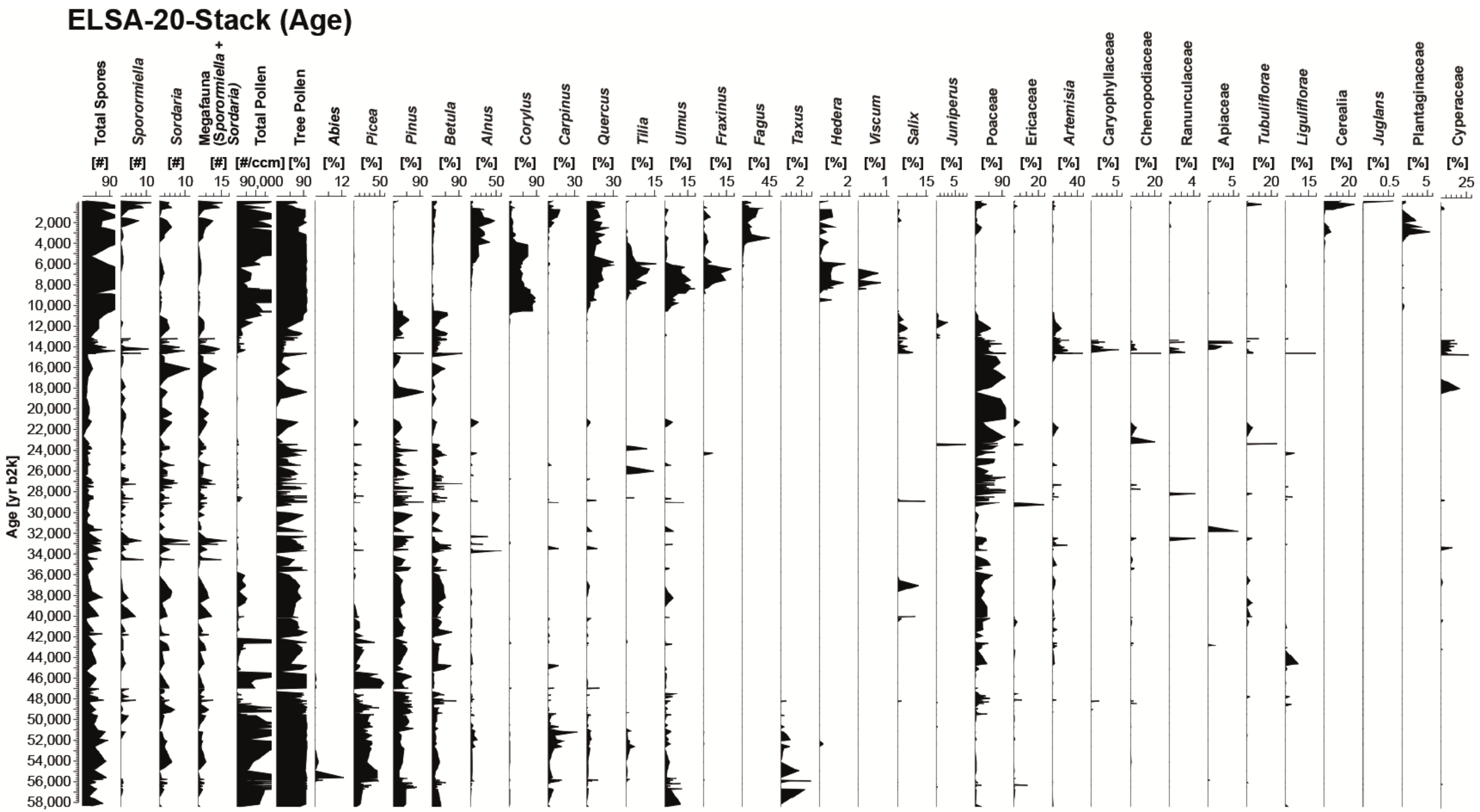

Supplementary Figure S9. Pollen concentrations and spore counts for the ELSA-20-Stack versus age. Composite of all counts from SMf, HM4, AU3, AU4.

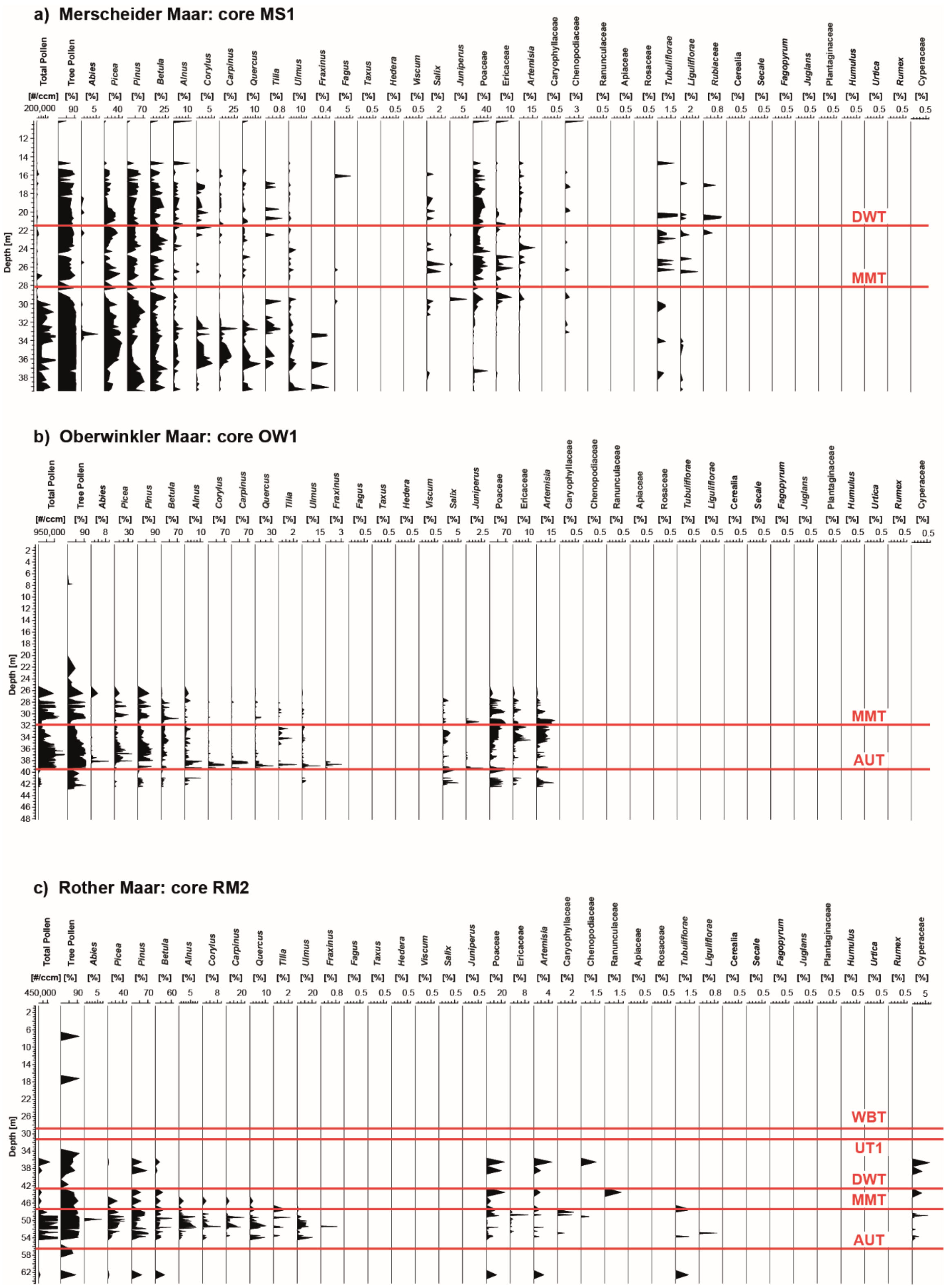

Supplementary Figure S10. Pollen concentrations and tephra layers for MIS3/2 in the ELSA cores from Merscheider Maar, Oberwinkler Maar, and Rother Maar versus age. These low resolution records show the early MIS3 spruce forest and the same tephra[40] as found in Auel[1] and Dehner Maar[2]. Accordingly, ELSA-20 ages were applied for the age model of these records.

Supplementary Tables S1 to S7

Supplementary Table S1. ELSA Stack Sample Information. For each stack sample there are given the respective core depth, sampled core, and age in yr b2k. For the samples that were analyzed for *n*-alkanes or Lithium isotopes, the respective values are documented.

Supplementary Table S2. All counts on pollen and spores from Holzmaar core HM4. Data are shown versus age and core depth.

Supplementary Table S3. All counts on pollen and spores from Auel infilled maar core AU4. Data are shown versus age and core depth.

Supplementary Table S4. All counts on pollen and spores from Holzmaar core HM4 and Auel infilled maar cores AU3,4 transferred to the ELSA-20-Stack. For each sample number we show the respective age and the drill core depth from which the samples were taken.

Supplementary Table S5. Pollen values from samples with at least 15 pollen grains from Merscheider Maar core MS1 versus age and core depth.

Supplementary Table S6. Pollen values from all samples from Rother Maar core RM2 versus age and core depth.

Supplementary Table S7. Pollen values from all samples from Oberwinkler Maar core OW1 versus age and core depth.
